# *Mycobacterium tuberculosis *from chronic murine infections that grows in liquid but not on solid medium

**DOI:** 10.1186/1471-2334-4-51

**Published:** 2004-11-17

**Authors:** Jasvir Dhillon, Douglas B Lowrie, Denis A Mitchison

**Affiliations:** 1 Department of Cellular and Molecular Medicine, St George's Hospital Medical School, London SW17 0RE, UK; 2 National Institute For Medical Research, Mill Hill, London NW7 1AA, UK

## Abstract

**Background:**

Old, stationary cultures of *Mycobacterium tuberculosis *contain a majority of bacteria that can grow in broth cultures but cannot grow on solid medium plates. These may be in a non-replicating, dormant growth phase. We hypothesised that a similar population might be present in chronic, murine tuberculosis.

**Methods:**

Estimates of the numbers of viable *M. tuberculosis*, strain H37Rv, in the spleens and lungs of mice in a 7-day acute infection and in a 10-month chronic infection were made by conventional plate counts and, as broth counts, by noting presence or absence of growth in serial replicate dilutions in liquid medium.

**Results:**

Plate and broth counts in 6 mice gave similar mean values in the acute infection, 7 days after infection. However, the broth counts were much higher in 36 mice with a chronic infection at 10 months. Broth counts averaged 5.290 log_10 _cfu /organ from spleens and 5.523 log_10 _cfu/organ from lungs, while plate counts were 3.858 log_10 _cfu/organ from spleens and 3.662 log_10 _cfu/organ from lungs, indicating that the total bacterial population contained only 3.7% bacilli in spleens and 1.4% bacilli in lungs, capable of growth on plates.

**Conclusion:**

The proportion growing on plates might be a measure of the "dormancy" of the bacilli equally applicable to cultural and animal models.

## Background

The organisms in a log phase, actively multiplying culture of *Mycobacterium tuberculosis *all grow well on plates and are estimated as colony forming units (cfu). However, cultures that have been grown undisturbed in the depths of liquid medium for 100 days contain a majority population which grows in liquid medium but is not able to form colonies on solid medium [1,2]. Since the bacilli in such cultures are hardly multiplying and have an uptake of [^3^H] uridine of only 15% of log phase cultures [1], they may be considered as dormant. A smaller population, that could grow on plates as well as in broth, possibly the survivors of the log phase bacilli, has also been demonstrated in these cultures. At the end of a 10-month mouse experiment on vaccines, we questioned whether the same two populations would be found, particularly in view of the evidence that there are similarities in gene expression patterns following adaptation to micro-aerophilic conditions in stationary cultures and exposure to NO in macrophages [3]. We therefore estimated populations present in the chronic infections in the organs of the 10-month mice both by conventional plate counts and by counts of the probable number of viable organisms obtained from serial dilutions in liquid medium. Similar counts were also set up, as a control, in a short-term acute infection in mice.

## Methods

### Culture media and bacteria

The media used were 7H9 liquid medium with 10% albumin, dextrose, catalase supplement and 0.05% Tween 80, and 7H11 agar medium with 10% oleic acid, albumin, dextrose, catalase supplement (Becton Dickinson, Oxford, UK). They were made selective by the addition of 100 μg carbenicillin, 200 U polymyxin B, 20 μg trimethoprim and 10 μg amphotericin B per ml (Mast, Bootle, UK) [4]. The two experiments both used 6-week, female Balbc mice, which were infected, with a mouse passaged H37Rv strain of *M. tuberculosis *suspended in 0.1% gelatin. The spleens and lungs were obtained at sterile autopsy and were homogenised as described elsewhere [5] in 5 ml water. From this suspension 100 μl amountsfrom the neat suspension and from serial 10-fold dilutions in 1 ml, were inoculated onto duplicate thirds of selective 7H11 medium plates. The number of colonies was counted after 3–4 weeks incubation at 37°C to give the plate count. A negative plate therefore had <25 cfu / organ. For the broth counts, serial 10-fold dilutions of the organ homogenate were made, in triplicate, in 1 ml amounts to 9 ml amounts of selective 7H9 broth with 0.05% Tween 80 in plastic 28 ml screw-capped bottle. In the acute infection, 10 serial dilution were set up, so as to obtain 30 tubes in all, while in the chronic infection 6 serial dilutions were set up yielding 18 tubes in all. These were incubated at 37°C and examined at 3 and 6 weeks and finally at 9 weeks for the characteristic growth of *M. tuberculosis*, namely a clear supernatant in undisturbed cultures with an upwards swirl of white growth on shaking,. Probable numbers of bacilli (pnb) per organ were obtained from a table of densities of organisms estimated by the dilution method [6]. Samples of the positive growth from 18 broth cultures were plated out on 7H11 medium.

### Acute infection experiment

Each of 6 mice was infected by the intravenous route with 200 μl of a suspension of a containing 2.6 × 10^6 ^cfu of the H37Rv strain. Plate and broth counts were carried out 7 days later.

### Chronic infection experiment

Each of 88 mice was infected by the intra-peritoneal route with 200 μl of a suspension containing 3.1 × 10^3 ^cfu of the H37Rv strain. Mice in our experiments are usually housed in an isolator, connected by a tunnel port to a Class 1 safety cabinet through which air from the environment is sucked. The intra-peritoneal route was chosen so that mice could be kept throughout the experiment in the isolator to prevent cross infection with mouse pathogens during exposure to the outside air in the Class 1 cabinet. One day after infection, samples of 6 mice yielded scanty or no colonies in plate counts of spleens and lungs. After a further 4 weeks these organ counts in 6 mice had risen to 2.24 × 10^4 ^in spleens and 1.15 × 10^4 ^in lungs. The mice were then divided into 6 experimental groups, 4 of which were vaccinated with various DNA vaccines over a 4-week period and 2 were unvaccinated controls. At 12 weeks after infection only 9 of 36 lungs and 18 of 36 spleens yielded positive growth on plates. At 10 months after infection, the 36 remaining mice were sacrificed, and plate and broth counts were set up on all, using dilutions estimated from a sample of 4 mice sacrificed 3 weeks earlier.

### Statistics

The results of the plate and broth counts were examined by 2-way analysis of variance using the Stata package, release 8 (Stata Corp., College Station, TX)

## Results

As the experimental vaccines appeared to have only small effects, which will be reported elsewhere, in the chronic infection model, the results in all 36 mice at 10 months are considered together. A typical broth count is shown diagrammatically in Table 1. Note that there were never any sporadic positive tubes in the no growth zones (inoculated in the example with 10^-4 ^or 10^-5 ^dilutions) of any set of broth cultures. The results in the 6 mice in the acute infection experiment and in 27 of the 36 mice in the chronic infection experiment that had assessable numbers of bacilli estimated as cfu by the plate method and as pnb by the broth method are set out in Table 2. In the analysis of variance of the acute infection counts, neither the variation between individual mice nor the difference between the counting methods was statistically significant. However, in the chronic infection, the broth counts were higher than the plate counts. In the spleens, the mean broth count was 5.290 log_10 _cfu / organ and the plate count was 3.858 log_10 _cfu /spleen. The difference between these counts is 1.432 log_10 _cfu / spleen (27-fold) so that, on the assumption that all bacilli that grew on plates also grew in broth, the bacilli capable of growing on plates comprised 3.7% (100/27) of the total count. In the lungs the mean broth count was 5.523 log_10 _cfu /lungs, about 73-fold higher than the plate count for the lungs. Thus the bacilli able to grow on plates comprised about 1.4% (100/73) of the total. The differences between individual mice were significant (p = 0.01) and highly significant between the counting methods (p < 0.001).

In the remaining 9 mice no colonies were obtained on the neat dilution plates in either the lungs alone or in both lungs and spleen (Table 3). However, broth counts were obtainable from both organs in all 9 mice, though their mean values were appreciably lower than those in Table 1. Where a comparison could be made between counting methods in the spleens of the 7 mice with colonies in plate counts, the means of the broth counts (4.347 log_10 _cfu / ml) were 19-fold higher than the corresponding plate counts (3.079 log_10 _cfu / ml, giving 5.3% of the total), in approximate agreement with the 27-fold (3.7%) estimate of the difference between broth and plate counts obtained from Table 1.

The following changes occurred in the broth counts during incubation. The counts between the 3-week and the 6-week readings increased on average in the lungs of each acute mice by 2.3 tubes and by 4.7 tubes in the spleens, and in each of the chronic mice by 2.0 tubes in lungs and 1.6 tubes in spleens. Thereafter, the increase from 6 weeks to 9 weeks was 1.5 tubes in the lungs of acute mice and 0.83 tubes in their spleens, while the increases in the lungs of chronic mice were 1.3 tubes and 1.0 tube in the spleens. Since an increase of 1 tube indicates a rise of about log_10 _0.8, that is about 12%, in the count, it is evident that counts increased during incubation, rapidly between 3 and 6 weeks and slowly between 6 and 9 weeks.

## Discussion

The chronic infection experiment was unusual in that the intraperitoneal infection in vaccinated mice led to trapping of the bacilli in the peritoneal cavity, so that few bacilli reached the organs, and thus the plate counts were sometimes negative, with a count of less than 25 cfu / organ. Variation, considerably greater than after intravenous or airborne infection, was also evident, the SD of the 4-week spleen counts, expressed as log10 cfu/organ, being 0.51 as compared to 0.23 for intravenous infection [5]. Evidence that growth in the broth cultures was *M. tuberculosis *was provided by the growth of typical colonies on 7H11 plates. That it was not due to sporadic contamination was shown by the usual complete absence of contamination in selective media, by a clear supernatant in the unshaken cultures and by the absence of any growth in the "no growth" zones of the broth cultures.

In log phase cultures (Hu Y-M, personal communication) and in the acute infection of mice, similar estimates of viable organisms were obtained in plate and broth counts However, our best estimate indicated that the bacilli in the chronic infections that would grow on plates was about 3.7% of the total population in spleens and 1.4% in lungs. This can be compared to the findings on a culture in 7H9 broth grown undisturbed for 100 days in the depths of liquid medium with caps screwed tightly on. In such a 100-day culture, population A, capable of growth in broth but not on plates, was estimated by broth dilution counts to be 7.60 log_10 _pnb / ml, while a smaller population B, that grew on plates, was estimated from parallel plate counts as 5.85 log_10 _cfu / ml.[1] Thus, population A was 1.75% of the total population. The corresponding estimates for a 30-day static culture were population A = 9.983 log10 pnb / ml and population B = 8.013 log10 cfu /ml, so that population B comprised 1.07% of the total population (Hu Y-M, personal communication).

These estimates suggest that the extent to which bacilli have gone into ill-defined dormancy might be measured as the proportion of a total bacterial population that can grow on plates. The lower this proportion, the greater the overall degree of "dormancy". Whatever, the theoretical significance of this simple technique for measuring dormancy, there is a practical implication for those undertaking long-term mouse experiments. Some end such an experiment with plate counts and others with culture in liquid medium. Those using only plate counts may be seriously underestimating the residual populations. It is also clear that there is much work to be done in seeing how various experimental conditions, such as the duration of the infection and the immune state of the mice, affect the ratio between broth and plate counts. Those exploring the development of new drugs need to know how these two populations respond to current anti-tuberculosis drugs and to novel drugs.

## Competing interests

The author(s) declare that they have no competing interests.

## Authors contributions

All three authors took part in the running of the experiments with JD contributing the most. DAM contributed the concept of parallel broth and plate counts.

## Pre-publication history

The pre-publication history for this paper can be accessed here:



## Figures and Tables

**Table 1:** Example of a broth count from a chronic infection mouse

	Tubes		
Dilution	1	2	3
10^-5^	0	0	0
10^-4^	0	0	0
10^-3^	0	+	0
10^-2^	+	+	+
10^-1^	+	+	+
10^0^	+	+	+

The mean fertile level (X) = no. of fertile cultures (10) / no. of cultures at each level (3). K = 0.760 from Table [6].

Log pnb = X-K + log5 (organ in 5 ml) = 3.272

**Table 2:** Plate and broth counts of *M. tuberculosis *in spleen and lungs of mice with acute and chronic infections

Infection	Mouse organ	Type of count*	Mean count	SD	95% confidence limits	
Acute (6 mice)	Spleen	Plate	7.070	0.214	6.845	7.295
		Broth	6.663	0.771	5.854	7.472
	Lungs	Plate	6.703	0.095	6.603	6.803
		Broth	6.609	0.421	6.167	7.050
Chronic (27 mice)	Spleen	Plate	3.858	0.882	3.509	4.207
		Broth	5.290	0.820	4.966	5.614
	Lungs	Plate	3.662	1.462	3.084	4.240
		Broth	5.523	0.939	5.152	5.894

* Plate counts are log_10 _cfu/organ. Broth counts are log_10 _probable number of bacilli/organ

**Table 3:** Plate and broth counts of *M. tuberculosis *in spleen and lungs of mice with chronic infections, when plate counts had no colonies

Organ with no colonies in plate counts*	No. of mice	Organ	Type of count^†^	Mean count	SD	95% confidence limits	
Spleen & lungs	2	Spleen	Broth	3.728	0.708	-2.638	10.094
		Lungs	Broth	4.247	1.428	-8.580	17.073
Lungs only	7	Spleen	Plate	3.079	0.960	2.192	3.968
			Broth	4.347	0.823	3.586	5.108
		Lungs	Broth	4.202	0.624	3.625	4.778

* A count of <1.40 log_10 _cfu/organ

^† ^Plate counts are log_10 _cfu/organ: broth counts are log_10 _probable number of bacilli/organ
